# Developmental environment mediates male seminal protein investment in *Drosophila melanogaster*

**DOI:** 10.1111/1365-2435.12515

**Published:** 2015-08-20

**Authors:** Stuart Wigby, Jennifer C. Perry, Yon‐Hee Kim, Laura K. Sirot

**Affiliations:** ^1^ Edward Grey InstituteDepartment of ZoologyUniversity of OxfordSouth Parks RoadOX1 3PSOxfordUK; ^2^ Jesus CollegeUniversity of OxfordTurl StreetOxfordOX1 3DWUK; ^3^ Department of BiologyCollege of WoosterWoosterOhio44691USA

**Keywords:** condition, density, development, ovulin, sex peptide, sexual selection

## Abstract

1. Males of many species fine‐tune their ejaculates in response to sperm competition risk. Resource availability and the number of competitors during development can also strongly influence sperm production. However, despite the key role of seminal proteins in mediating reproductive processes, it is unclear whether seminal protein investment is dependent on the developmental environment.
2. We manipulated the developmental environment of *Drosophila melanogaster* by rearing flies at low and high density. As expected, this resulted in large and small (i.e. high and low condition) adult phenotypes, respectively.
3. As predicted, large males produced more of two key seminal proteins, sex peptide (SP) and ovulin, and were more successful at obtaining matings with both virgin and previously mated females. However, there was only a weak and non‐significant trend for large males to transfer more absolute quantities of SP at mating, and thus, small males ejaculated proportionally more of their stored accessory gland SP resources.
4. Males transferred more receptivity‐inhibiting SP to large females. Despite this, large females remated more quickly than small females and thus responded to their developmental environment over and above the quantity of SP they received.
5. The results are consistent with two non‐mutually exclusive hypotheses. First, flies might respond to condition‐dependent reproductive opportunities, with (i) small males investing heavily in ejaculates when mating opportunities arise and large males strategically partitioning SP resources and (ii) small females remating at reduced rates because they have higher mating costs or need to replenish sperm less often.
6. Second, flies may be primed by their larval environment to deal with similar adult population densities, with (i) males perceiving high density as signalling increased competition, leading small males to invest proportionally more SP resources at mating and (ii) females perceiving high density as signalling abundant potential mates, leading to a higher sexual receptivity threshold.
7. Thus, by influencing the mating frequencies of both sexes, as well as the quantity of seminal proteins produced by males and received by females, the developmental environment is likely to have far‐reaching and sex‐specific consequences for sexual selection and sexual conflict.

## Introduction

Males have been traditionally viewed as having fixed ejaculate strategies, resulting from strong directional selection to maximize gamete transfer at each copulation. However, this view has changed as a result of evidence documenting finely tuned plasticity in how males allocate sperm in response to the social and sexual environment in many taxa (Simmons 2001; Wedell, Gage & Parker 2002; Birkhead, Hosken & Pitnick 2008). Recently, theoretical and empirical studies have begun to reveal similar plasticity in male investment in non‐sperm components of the ejaculate (Hodgson & Hosken 2006; Cameron, Day & Rowe 2007; Wigby *et al*. 2009; Alonzo & Pizzari 2010; Perry & Rowe 2010; Fedorka, Winterhalter & Ware 2011; Sirot, Wolfner & Wigby 2011; Perry, Sirot & Wigby 2013). For example, male *Drosophila melanogaster* are able to adjust the titres of individual seminal fluid proteins (Sfps) transferred to females in response to their mating status, potentially exploiting the effects of Sfps transferred by a female's previous mates (Sirot, Wolfner & Wigby 2011). This remarkable degree of plasticity is consistent with current understanding of Sfps as key mediators of male reproductive success and intersexual conflict (Sirot *et al*. 2014).

Despite their key role, the extent of Sfp plasticity with respect to other social and ecological factors remains little known. For example, we know little about Sfp plasticity in response to the developmental environment. Greater competition during development may limit resources and influence adult condition (defined as the pool of resources an individual has available to invest in trait expression; Rowe & Houle 1996). Both theoretical and empirical studies suggest resource‐dependent plasticity (i.e. condition dependence) in the expression of sexually selected traits in general (Iwasa & Pomiankowski 1991; Rowe & Houle 1996; Cotton, Fowler & Pomiankowski 2004). Sperm quantity and quality are also sensitive to resource abundance in many taxa (Teletchea *et al*. 2009; Perry & Rowe 2010; Lewis, Sasaki & Miyatake 2011). Yet, there is currently limited understanding of condition dependence in Sfp production and allocation. Moreover, it is not clear that the prediction of heightened condition dependence should apply to Sfps. The prediction of heightened condition dependence in sexually selected traits assumes that high‐condition males have lower marginal costs of trait production than low‐condition males, and traits should also be subject to directional selection, for example for increased quantity (Grafen 1990; Lachmann, Szamado & Bergstrom 2001). However, there is currently limited data available on the costs of Sfp production (Perry, Sirot & Wigby 2013; Friesen *et al*. 2015; Sirot & Wolfner 2015). Furthermore, traits involved in post‐copulatory interactions are often subject to strong stabilizing selection (e.g. for species recognition; Eberhard *et al*. 1998; Simmons *et al*. 2009; Simmons 2014) and may not be subject to positive directional selection if there is a threshold above which males gain no additional benefit (e.g. as with female responses to ‘sex peptide (SP)’ in *D. melanogaster*; Schmidt *et al*. 1993). Thus, we currently do not know the extent to which the prediction of heightened condition dependence should apply to Sfps. Furthermore, an increased density of competitors during development – associated with resource limitation – could also signal high reproductive competition during adulthood. Individuals developing in environments of high resource competition might optimize their reproductive strategies for higher reproductive competition during adulthood (Gage 1995; Schärer & Ladurner 2003; Lemaitre *et al*. 2010). However, we currently know little about how such developmental factors influence the production and transfer of male Sfps or female Sfp‐mediated behaviours in *Drosophila*.

Here, we experimentally investigated developmental environment‐mediated plasticity in male Sfp production and transfer in *D. melanogaster* and its consequences for female behaviour, by varying population density (high or low) during larval development. Larval density and nutrition regulate adult body size, which is associated with male success in pre‐ and post‐copulatory sexual competition (Miller & Thomas 1958; Lefranc & Bundgaard 2000; Bangham, Partridge & Chapman 2002; Pitnick & García–González 2002) and female attractiveness and fecundity (Long *et al*. 2009; Lüpold *et al*. 2011). Hereon, for conciseness and consistency with previous literature, we use ‘small’ and ‘large’ to refer to flies reared at high and low larval density environments, respectively. We focused on two key Sfps: ovulin (OV), a protein that increases ovulation rate (Herndon & Wolfner 1995), and SP (Chapman *et al*. 2003; Liu & Kubli 2003), a multifunctional protein whose effects include inhibiting remating and promoting egg production (reviewed in Ravi Ram & Wolfner 2007). We tested for differences in the quantity of these two Sfps produced and transferred based on the size of the male, the size of his mating partner and the size of rival males. To set developmental environment‐dependent Sfp transfer in context, we conducted behavioural assays to test whether male mating opportunities depended on male size, and whether female remating behaviour depended on female size and the size of a female's past and present mates.

Our results provide evidence for developmental environment‐dependent Sfp allocation by males, as well as unexpected female remating patterns. Small males, despite having reduced ejaculate resources relative to large males, invested proportionally larger amounts of Sfps in matings. This pattern is consistent with strategic allocation of Sfp reserves because, relative to males developing in low‐density environments, males developing in a high‐density environment may have more competitors (Gage 1995; Schärer & Ladurner 2003; Lemaitre *et al*. 2010) and fewer mating opportunities due to their size (small *D. melanogaster* males have lower mating success than large males; Partridge, Ewing & Chandler 1987; Pitnick 1991). Males, irrespective of their own developmental environment, invest more SP in large females. Large females remate more frequently, despite receiving larger quantities of receptivity‐inhibiting SP, suggesting that, for females, their developmental environment regulates remating, over and above the receipt of receptivity‐inhibiting substances from males.

## Materials and methods

### Stocks

We used a Dahomey wild‐type stock of *D. melanogaster* (Wigby *et al*. 2011) maintained at 25 °C on a 12:12 L:D cycle. All flies used in the experiments were between 3 and 10 days post‐eclosion. Within experiments, flies were age‐matched to within 1–2 days, and virgins at the start of experiments, unless stated otherwise. All experimental matings and rematings were ‘no‐choice’ – one female was presented with one male.

### Rearing Large and Small Flies

To produce adult flies of large and small body sizes, we manipulated egg and, therefore, larval density over four experiments. Manipulating larval density is a commonly used technique for manipulating adult size, which alters resource availability per larvae while keeping the food type constant across treatments (e.g. Pitnick 1991; Lefranc & Bundgaard 2000; Pitnick & García–González 2002; Byrne & Rice 2006; Amitin & Pitnick 2007; Lüpold *et al*. 2011). Varying larval density may additionally alter the perception of future adult reproductive competition (e.g. Gage 1995). Experiments 1 and 2 were conducted at the College of Wooster, USA, and experiments 3 and 4 were conducted at the University of Oxford, UK. Eggs were collected from 250‐mL glass bottles (for experiments 1 and 2, see below) or a population cage (for experiments 3 and 4) using grape juice agar plates with a drop of live yeast paste. Flies were grown, and experiments conducted, using the standard fly food for each laboratory, to which each laboratory's fly stocks were adapted. To produce small flies for experiments 1 and 2, we placed approximately 400 eggs on 1–2 mL of dextrose–yeast food medium in 36‐mL vials. To produce large flies, we placed approximately 200 eggs on 50 mL of the same food medium in 250‐mL bottles. We followed the same procedure for experiments 3 and 4 but used a sugar–yeast–maize–molasses food medium (Lewis 1960). Although different foods were used in experiments 1 and 2 from experiments 3 and 4, the effects of manipulating density on adult body size were similar (see Results), and thus, the phenotypic effects are qualitatively comparable. Using larger containers to rear the low‐density treatment allowed us to keep the absolute population size within each container of the same order of magnitude and thus ensure that both low‐ and high‐density groups were grown in groups of several hundred flies. Subsequently ‘large’ refers to flies reared at low density and ‘small’ refers to flies reared at high density. Adult flies were collected as virgins within 8 h of eclosion and separated into same‐sex vials (10–20 flies per vial) containing food medium sprinkled with live yeast. All experiments were conducted in vials containing food medium and live yeast, unless specified otherwise.

### Quantification of Sfps

ELISAs were used to quantify SP and OV in male accessory glands (AGs) and reproductive tracts of mated females following methods previously described (Sirot *et al*. 2009; Wigby *et al*. 2009). A Molecular Devices VERSAmax plate reader (Molecular Devices, LLC, Sunnydale, CA, USA) was used to determine the optical density in the final step of the process.

### Test of Interference of Female Tissue with Detection of Sfps in ELISAs

We first tested whether our ability to detect a standard amount of SP or OV was different for large and small female reproductive tracts due to potential interference from the female tissue in the ELISA samples. We prepared samples of reproductive tracts from individual virgin large and small females, spiked each sample with a consistent amount of male accessory gland tissue (1/8th male accessory gland equivalent per 50 μL sample) and measured SP and OV levels in each sample using ELISAs. We found no significant effect of female size on the amount of SP detected (mean accessory gland standard equivalent ± SE, large = 0·159 ± 0·005, *N* = 9, small = 0·161 ± 0·0023, *N* = 9; *t*_16_ = 0·145, *P* = 0·89). However, we detected significantly less OV in large female samples relative to small female samples (mean accessory gland standard equivalent ± SE, large = 0·147 ± 0·009, *N* = 10; small = 0·171 ± 0·005, *N* = 10, *t*_18_ = 2·31, *P* = 0·033). We therefore analysed the effects of both male and female sizes on SP transfer, but only used OV data to test for male size effects while keeping female size constant; that is, we did not statistically compare large vs. small females for OV, to avoid potential confounds of varying female tissue quantity among large and small females on OV detection, but we did compare effects of male size on OV transfer separately for the two female size classes (i.e. large vs. small males mated to small females, and large vs. small males mated to large females; see Experiment 1).

### Seminal Fluid Protein Production and Allocation to Females

We measured Sfp production by males and allocation to virgin females in single mating trials in experiments 1 (both SP and OV) and 2 (SP only) described below. In both experiments, virgin females were anesthetized on ice and placed individually in vials 1 day before the experiment began. Large and small females were randomly assigned to each size or treatment group of male (detailed below). On the morning of the experiment, males from the assigned size or treatment group were singly aspirated into each female vial (i.e. one male was placed with one female within each vial) and a single mating was allowed to occur. We used continuous scans to record latency until mating and mating duration until the mating finished. Flies were given up to 3 h to mate, and any non‐mating flies were discarded. At 25 min after the start of single mating, females were flash‐frozen in liquid nitrogen and stored at −80 °C until dissection for measurement of Sfp transfer by ELISAs (as in Wigby *et al*. 2009; Sirot, Wolfner & Wigby 2011).

### Experiment 1: Effects of Developmental Environment on Adult Sfp Production and Transfer

To test whether the developmental environment influences the quantity of Sfps produced by males and transferred during mating, we (i) measured Sfps in the accessory glands of large and small males, and (ii) paired large and small males and females in all four possible size combinations (i.e. large–large, large–small, small–large and small–small) and measured the quantity of Sfps present in female reproductive tracts after a single copulation. The experiment was replicated in two blocks (*N* = 67–76 per male–female combination). A subset of males was flash‐frozen for dissection and ELISAs on SP and OV, at 3 days (block 1) or 6 days (block 2) after mating (*N* = 18–20 per size class). A further subset of flies was weighed to test for differences in mass between large and small flies. Weighed flies were between 5 and 7 days post‐eclosion; females were virgins (groups of 5 flies, *N* = 6 large and 9 small groups) that were not used in the mating experiments, and males were experimental males 30 h post‐mating (groups of 5 flies, *N* = 23 large and 24 small groups).

### Experiment 2: Effects of Rival Male Size on SP Production and Transfer

Next, we tested whether males change their mating patterns and SP allocation depending on the size of rival males, while keeping female size constant. To do this, we measured SP transfer to large females by large or small males that had previously been housed with either large or small rival males. We also estimated the relative SP depletion of males from a single mating to provide an additional measure of the quantity of SP transferred at mating. We placed large and small males in all pairwise combinations into vials (i.e. two males per vial: large–large, large–small or small–small) for 39 h prior to the experiment. On the morning of the experiment, a randomly chosen subset of males was retained as virgins to measure SP production. We placed single experimental males into vials containing single large females (note, no small females were used in this experiment). Mated females and males were flash‐frozen for ELISAs 25 min after the start of mating. ELISAs were performed on 46–56 females and 25–26 males per rival combination. Virgin males maintained with rivals were frozen concurrently with mated males. ELISAs were performed on *N** = ***23–24 virgin males held with a same‐sized rival (i.e. large–large or small–small).

### Mating Behaviour

To investigate whether Sfp production and transfer was associated with male ability to obtain mates we tested whether male and female sizes influenced (i) latency to mate with virgin or previously mated females and (ii) male courtship and female rejection rates. We measured latency to mating in two experiments using virgin females (experiments 1 and 2 described above) and latency to mating and proportion of females remating in two separate experiment using females both as virgins and subsequently as mated females (experiment 3 and 4 described below). To determine whether differences in remating propensity were driven by changes in male or female behaviour, in Experiment 4, we conducted close observations of male courtship of previously mated females and female resistance behaviours.

### Experiments 3 and 4: Mating, Remating, Courtship and Rejection Behaviour

For both experiments 3 and 4, large and small virgin females were singly aspirated into vials 1 day prior to matings. To measure the effect of male and female sizes on latency to mating, a single large or small virgin male was then aspirated into each vial at lights on, and latency to mating and duration of mating were recorded. Males were removed from the vials immediately after copulation. On the following day (22***–***24 h later), the mated females were randomly assigned either a large or small virgin male. We tested whether the propensity of females to remate depended on (i) the size of their previous mate, (ii) the size of the female and (iii) the size of their current potential partner.

In Experiment 3, we measured the latency to remating of females, whereas in Experiment 4, we focussed on measuring male courtship and rejection behaviour by females (in addition to recording rematings), which required closer observation. Thus, in Experiment 3, the fresh male was added to the female's vial, but in Experiment 4, each female was aspirated into an ‘observation chamber’ (a small Petri dish of 2 cm diameter containing moistened filter paper and a blob of live yeast paste) at lights on, and the assigned male was added to the chamber. Using the chambers in Experiment 4 allowed us to observe fly behaviour more closely than is possible when flies are in vials. Latency until mating and duration of mating were recorded in both experiments (for 6 h in Experiment 3 and 3 h in Experiment 4). Additionally, in Experiment 4, male courtship and female behaviours were observed by spot sampling every 10 min over 3 h or until mating. The male courtship behaviours recorded were singing, chasing, licking and attempting copulation. The female resistance behaviours were running away from pursuing males, flying away, ovipositor extrusion and wing folding (reviewed in Yamamoto & Koganezawa 2013).

To obtain a sufficient sample size, Experiment 3 was conducted in 3 blocks, and each block took place over 3 days. Flies tested on ‘day 1’ were therefore 1 day younger than those tested on ‘day 2’, which in turn were 1 day younger than those tested on ‘day 3’. The time between 1st and 2nd matings was always 1 day. Total sample size over all blocks was 363 females (*N* = 15***–***18 females per treatment for blocks 1 and 2 and 10–12 females per treatment for block 3; overall *N* = 42–48 for each combination of 1st and 2nd male and female sizes).

Experiment 4 was performed over 2 days, with the result that flies tested on the second day were 1 day older than flies tested on the first day. We tested 80 females in total (20 for each combination of large and small males and females). We weighed a subset of flies (*N* = 16 per size, per sex).

### Statistical Analysis

We tested for an effect of larval density on adult fly mass using anova, separately for each sex and separately for the Sfp and behavioural experiments. To analyse latency to first mating (i.e. where females were virgin, so virtually all mated) and mating duration data, we used linear mixed models. For latency to remating, we conducted a proportional hazards survival analysis, to account for non‐remating females (overall approximately half of the females did not remate). We analysed Sfp data, using linear mixed effect models. ELISA plate was entered as a random factor when there were more than 6 levels (Experiment 2, female Sfp analysis) or as a fixed effect in linear models when there were 5 or fewer levels (all other analyses) (Bolker *et al*. 2009). The proportion of courtship rejected was calculated as the number of rejection events a female performed divided by the total number of courtship events she received. The proportion of time spent courting, proportion of courtship rejected and proportion of pairs remating (Experiment 4) were analysed using generalized linear models (GLMs) with quasi‐binomial error distributions, to account for overdispersion. Where necessary, latency, mating duration and Sfp data were Box‐Cox transformed to improve normality. When significant, block, day and ELISA plate were retained in models (reported in Data S1, Supporting information), and where experiments were conducted over multiple days within blocks (experiments 3 and 4), we included ‘day’ as a fixed factor to account for the changes in fly age over time. In Experiment 1, two long mating duration outliers were removed (one from the large male/small female treatment, the other from the small male/large female treatment; Grubb's test, *P* < 0·0001). Where data were obtained over replicate blocks, we included block as a fixed factor in models, because the number of levels was always <6 (Bolker *et al*. 2009). Where *P*‐values were combined across multiple experiments, we used Fisher's method (Sokal & Rohlf 1995). Data were analysed using jmp v9, SAS Institute, Inc., Cary, North Carolina, USA (anovas, LMs and proportional hazards) and r 1.40; R Core Team (2013) (GLMs and GLMMs). Models were simplified by removing non‐significant factors to obtain the minimum adequate model. For the GLMs and GLMMs, the significance of factors was assessed by comparing models with and without the factor.

## Results

### Larval Density Effects on Body Size

As expected, higher density larval environments resulted in highly significantly smaller adult flies for both sexes [mean mass (mg) ± SE: Experiment 1: females, large = 1·50 ± 0·04, small = 0·58 ± 0·03, *F*_1,13_ = 329·0; males, large = 0·83 ± 0·03, small = 0·50 ± 0·03, *F*_1,45_ = 210·4; Experiment 4: females, large = 1·60 ± 0·06, small = 0·814 ± 0·08, *F*_1,30_ = 272·1; males, large = 0·87 ± 0·03, small = 0·60 ± 0·06, *F*_1,30_ = 85·7; *P* < 0·0001 for all within‐sex comparisons].

### Seminal Fluid Protein Production

**Figure 1:**
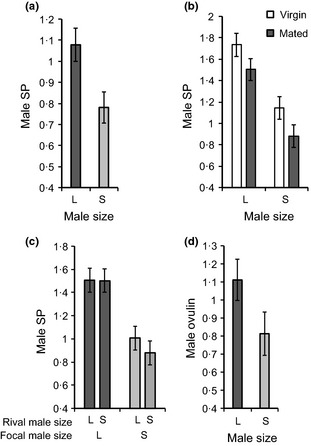
Sfp production by large (L) and small (S) males (mean ± SE). Quantities shown are relative to a male accessory gland standard. Sex peptide in male accessory glands (a) several days (3–6) after mating (Experiment 1), (b) immediately after mating or as virgins (Experiment 2), and (c) in response to large or small rival males (Experiment 2). (d) ovulin production in mated (3–6 days post‐mating) males (Experiment 1).

The quantity of SP present in male accessory glands was significantly higher for large compared to small males. This was true for males several days after mating (Experiment 1: *F*_1,35_ = 5·86, *P* = 0·021; Fig. 1a), immediately after mating (Experiment 2: *F*_1,95_ = 31·91, *P* < 0·0001; Fig. 1b), and as virgins (Experiment 2: *F*_1,45_ = 19·45, *P* < 0·0001; Fig. 1b). There were no significant effects of rival male size on SP (Experiment 2; *F*_1,94_ = 0·50, *P* = 0·48; Fig. 1c) and no interaction between focal male size and rival male size (*F*_1,93_ = 0·52, *P* = 0·47; Fig. 1c). Similar to SP, there was a trend for higher OV in large compared with small males (Experiment 1: male size *F*_1,38_ = 3·91, *P* = 0·055; Fig. 1d).

### Seminal Fluid Protein Allocation to Females

#### Absolute quantity of Sfps transferred during mating

**Figure 2:**
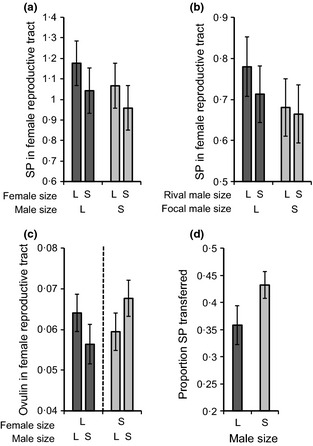
Sfp transfer to females (mean ± SE). L, large, S, small. Quantities shown are relative to a male accessory gland standard. Sex peptide (SP) present in the reproductive tracts of females (a) in response to male and female sizes (Experiment 1) and (b) in response to male size and the size of male rivals (Experiment 2). (c) Ovulin present in female reproductive tracts in response to male size (Experiment 1). These data were analysed separately (denoted by dashed line) for large and small females (see Materials and methods). (d) Estimated proportion of SP reserves transferred to females by large and small males (Experiment 2).

We found significantly more SP in the reproductive tracts of large females than small females (Experiment 1; *F*_1,278_ = 12·00, *P* = 0·0006; Fig. 2a). We found non‐significant trends for large males to transfer more SP than small males in both experiments 1 and 2 [Experiment 1: *F*_1,278_ = 3·41, *P* = 0·069; Fig. 2a; Experiment 2 (note, analysis using mixed model: see Materials and methods): focal male size, $\chi_{1}^{2}$ = 2·06, *P* = 0·15; Fig. 2b] and when the *P*‐values from the two experiments were combined (*P* = 0·0563) (Sokal & Rohlf 1995). No other tested effects were significant or marginal (Experiment 1: male size x female size interaction, *F*_1,276_ = 0·30, *P* = 0·58; mating duration, *F*_1,277_ = 0·50, *P* = 0·48; Experiment 2: rival size, $\chi_{1}^{2}$ = 1·05, *P* = 0·30; mating duration, $\chi_{1}^{2}$ = 1·19, *P* = 0·28; male size x rival size interaction, $\chi_{1}^{2}$ = 0·19, *P* = 0·66).

In preliminary experiments (see Materials and methods), we found that female size affected the detectability of OV in the female reproductive tract. As a result, we restricted our analysis of the effect of male size on OV transfer to within female size classes. We found no significant effect of male size on OV transfer within either female size class (Fig. 2c; within large females: *F*_1,130_ = 3·13, *P* = 0·079; within small females: *F*_1,139_ = 0·80, *P* = 0·372). Mating duration was positively associated with OV levels in both female size classes (large females: *F*_1,131_ = 8·49, *P* = 0·004; small females: *F*_1,140_ = 9·75, *P* = 0·002). Mating duration itself was influenced by an interaction between male and female sizes (Data S1; Fig. S1).

#### Proportion of sex peptide transferred during mating

Because large males produced more SP, but only showed a non‐significant trend to transfer more SP, we tested the hypothesis that large males transferred a lesser proportion of their SP reserves than small males. First, we compared the average amount of SP in the accessory glands of virgin and mated males for the two size classes. On average, the accessory glands of large males contained 13% less SP after mating, compared with 23% less SP for the accessory glands of small males after mating (Experiment 2, Fig. 1b), suggesting that small males transfer proportionally more SP at mating – relative to their initial SP reserves – than large males.

To test this hypothesis further, we used measures of the SP present in individual male accessory glands immediately after mating and SP present in the reproductive tract of their mates. This allowed us to calculate the proportion of SP transferred to females (Experiment 2), as the quantity of SP detected in the female reproductive tract divided by the summed quantities of SP present in the male accessory glands and the female reproductive tract for each mating pair. Consistent with our initial finding, the proportion of accessory gland SP transferred at mating was significantly higher for small males compared with large males (*F*_1,99_ = 4·95, *P* = 0·028; Fig. 2d). No other effects were significant (rival size, *F*_1,89_ = 0·13, *P* = 0·72; interaction between focal male size and rival size, *F*_1,88_ = 0·056, *P* = 0·82; mating duration, *F*_1,90_ = 0·37, *P* = 0·54).

These results demonstrate that despite producing more SP than small males, large males transfer proportionally less of their stored SP to females during their first mating. Further, males transfer more SP to large than to small females during their first mating. To test whether these results are consistent with the hypothesis of strategic allocation of Sfps based on condition‐dependent mating opportunities, we next tested whether

1. Large males are more successful in mating with females than small males, which would provide an advantage to ‘saving’ SP for future matings and
2. Large females are less likely to remate than small females, as expected because they receive more SP in first matings.

### Male and Female Mating Rates

#### Virgin females

**Figure 3:**
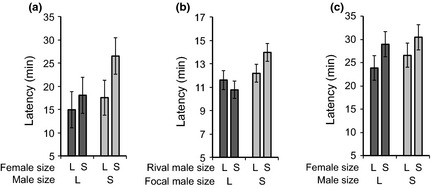
First mating (i.e. virgin) latency to mating (mean ± SE) in response to male and female sizes (a, c) and the size of male rivals (b). (a) Experiment 1, (b) Experiment 2, (c) Experiment 3.

Combined *P*‐values across experiments (Sokal & Rohlf 1995) revealed that, overall, small males (*P* = 0·019) and small females (*P* = 0·004) took significantly longer to mate than large males and females (males: Experiment 1, *F*_1,358_ = 3·01, *P* = 0·084; Experiment 2: *F*_1,214_ = 6·18, *P* = 0·014; Experiment 3, *F*_1,357_ = 0·59, *P* = 0·44; females: Experiment 1, *F*_1,359_ = 7·92, *P* = 0·005; Experiment 3, *F*_1,358_ = 2·30, *P* = 0·13; Fig. 3a–c). There were no significant interactions between male and female sizes (Experiment 1, *F*_1,357_ = 5·45, *P* = 0·49; Fig. 3a; Experiment 3, *F*_1,356_ = 0·05, *P* = 0·83; Fig. 3c) or between focal male and rival male size, and no rival male size effect (Experiment 2, interaction, *F*_1,212_ = 0·38, *P* = 0·54, rival male size, *F*_1,213_ = 0·44, *P* = 0·51; Fig. 3b).

#### Remating in previously mated females

**Figure 4:**
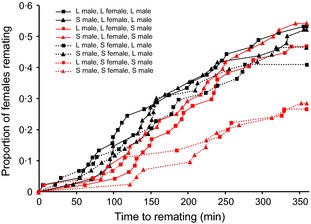
Remating behaviour of large and small males and females. Large or small females were mated first to either a large or small male and then exposed for remating to either a large or small male. Squares = large first male, triangles = small first male; solid line = large female, dotted line = small female; black = large second male; red = small second male.

Remating rates were significantly higher for large females [$\chi_{1}^{2}$ = 8·68, *P* = 0·0032, risk ratio (large : small) ± 95% CI = 1·61, 1·17–2·22] and large males ($\chi_{1}^{2}$ = 3·99, *P* = 0·046, risk ratio = 1·37, 1·01–1·88; Fig. 4). However, remating rates were not affected by first male size ($\chi_{1}^{2}$ = 0·25, *P* = 0·61, risk ratio = 0·92, 0·68–1·26; Fig. 4), or by interactions between first male size, second male size and female size (all combinations $\chi_{1}^{2}$ < 2·64, *P* > 0·1).

#### Courtship and rejection

Neither male nor female size significantly influenced male courtship of or rejection by previously mated females (Experiment 4: mean proportion of time courting ± SE; L male L female, 0·623 ± 0·083; L male S female, 0·581 ± 0·083; S male L female, 0·599 ± 0·083; S male S female, 0·601 ± 0·083; female size, *F*_1,78_ = 0·04, *P* = 0·84, male size, *F*_1,77_ = 0·00, *P* = 0·98; interaction, *F*_1,76_ = 0·10, *P* = 0·55; mean proportion of courtship rejected by females ± SE; L male L female, 0·920 ± 0·023; L male S female, 0·956 ± 0·024; S male L female, 0·926 ± 0·024; S male S female, 0·966 ± 0·024; female size, *F*_1,70_ = 2·44, *P* = 0·12; male size, *F*_1,68_ = 0·11, *P* = 0·74; interaction, *F*_1,67_ = 0·03, *P* = 0·86; Fig. S2).

## Discussion

Our results show that changes in larval density for both males and females can have far‐reaching consequences for adult male seminal protein production and transfer, and for female remating patterns. The data are broadly consistent with strategic responses of males to their own condition and mating rates, as well as to the condition of their mates, whereby (i) small, low‐condition males have limited ejaculate resources and are slower to obtain matings, but allocate relatively more Sfps during copulation and (ii) males allocate more SP to large, high‐condition females. An alternative, non‐mutually exclusive explanation for this pattern is that small males allocate relatively more of their SP resources because they perceive higher male–male competition (including sperm competition) from having encountered a higher density of competitors during larval development. However, despite large females receiving more SP than small females, large females nonetheless remate sooner, demonstrating that larval density has a potent influence on remating rates, over and above SP quantities.

Theory predicts that when high‐condition males have lower marginal costs of ejaculate production, they should allocate more ejaculate at mating (Parker 1990; Tazzyman *et al*. 2009). Although we found that small males had reduced Sfps present in their accessory glands, there was not strong evidence that they transferred reduced quantities of Sfps to females at mating: there were only non‐significant trends for the quantities of Sfps detected in females after mating, and those lost from male accessory glands during mating, to be higher for large than small males. This result is supported by the post‐mating receptivity of females in our study, which did not depend on the size of their previous mate, as would be expected if females received similar quantities of receptivity‐inhibiting SP from large and small males. Previous studies have found that female *D. melanogaster* tend to have higher fecundity in the day following a mating with a small compared with a large male (Pitnick 1991; Lefranc & Bundgaard 2000; Imroze & Prasad 2011). These results have been attributed to difference in the ejaculate composition of small and large males. If this was the case, and if Sfps act in a linearly dose‐dependent manner, we would expect smaller males to transfer more OV – which enhances fecundity in the first 24 h (Herndon & Wolfner 1995) – compared with larger males. Yet, we found no effect of male size on the amount of OV transferred to females. Thus, our data suggest that male size has relatively little impact on the absolute quantity of Sfps that males transfer and no evidence of an effect on the post‐mating receptivity response they induce in females in *D. melanogaster*.

However, we found that small males transferred a greater proportion of their stored Sfp reserves at mating, as compared with large males, which is consistent with hypotheses based on ejaculate prudence (Wedell, Gage & Parker 2002) and mating opportunities (Pitnick 1991). Females mate faster with large than with small males (the present study and Pitnick 1991), and small males lose out in competition with large males (Partridge, Ewing & Chandler 1987), so it may pay small males to invest heavily in any matings achieved, as has been argued for similar patterns found in garter snakes (*Thamnophis sirtalis parietalis*; Friesen *et al*. 2015). In contrast, large males that may have multiple mating opportunities might risk ejaculate depletion if they do not partition their ejaculate (Hihara 1981; Linklater *et al*. 2007; Sirot *et al*. 2009). Thus, large males may benefit from strategically transferring proportionally less of their ejaculate at mating, allowing them to conserve ejaculate resources for future copulations. However, our data are also consistent with the hypothesis that males allocate a fixed quantity of Sfps at mating independent of their own body size, Sfp resources and mating opportunities. A further, non‐mutually exclusive possibility is that males from high‐density larval environments increase the relative allocation of Sfps because they perceive high larval density as signalling a high risk of sperm competition in adulthood (Gage 1995; Schärer & Ladurner 2003; Lemaitre *et al*. 2010). This hypothesis could be further tested by varying adult male condition using methods that do not involve changes in population density, for example, larval food dilution (Amitin & Pitnick 2007; McGraw *et al*. 2007; Zikovitz & Agrawal 2013).

Males did not adjust Sfp allocation in response to the size of their rival, suggesting that males do not apply a context‐dependent rule depending on the relative quality of local competitors. This result is perhaps surprising given that exposure to rival males affects mating and ejaculate investment behaviours (Bretman, Fricke & Chapman 2009; Wigby *et al*. 2009; Bretman *et al*. 2010; Garbaczewska, Billeter & Levine 2013). Future work should explore other factors that may influence plastic Sfp allocation patterns, including immediate variation in mating opportunities (e.g. sex ratio (Linklater *et al*. 2007).

In our study, we found increased levels of SP after mating in large females compared with small females. This finding is consistent with the prediction that males strategically invest more in ejaculates transferred to females of higher reproductive value (Wedell, Gage & Parker 2002), given that female fecundity increases with body size in *D. melanogaster* (Lefranc & Bundgaard 2000; Pitnick & García–González 2002; Long *et al*. 2009). Our finding is also consistent with previous research showing that male *D. melanogaster* allocate more sperm to large females (Lüpold *et al*. 2011). Sperm numbers are sensitive to size and condition in other species (O'Dea, Jennions & Head 2014), but whether this is true for *D. melanogaster* is not known, nor to what extent sperm and Sfps can be adjusted independently. Previous studies suggest that rapid successive matings deplete Sfps to a greater extent than sperm (Hihara 1981; Linklater *et al*. 2007), but this question warrants direct investigation. We were unable to test impact of female size on OV transfer because the increased quantity of female tissue from large females interfered with our ability to detect OV (see Materials and methods). Future studies could test for such differential OV transfer by measuring the loss of OV from male accessory glands at mating.

Given that large females received more receptivity‐inhibiting SP, and likely larger quantities of sperm (Lüpold *et al*. 2011) on which SP is carried (Peng *et al*. 2005), we would expect large females to show reduced post‐mating receptivity. However, we found that large females remated more rapidly than small females, consistent with previous reports (Amitin & Pitnick 2007). A possible explanation is that the effects of SP are diluted in large females due to their increased body volume. In our experiments, large females were approximately twice the mass of small females, whereas the quantity of SP in large females’ reproductive tracts was approximately 10% higher than in small females. Thus, a lower concentration of SP in large females’ reproductive tract and haemolymph might have resulted in reduced activation of the SP receptor and hence reduced post‐mating refractoriness. Large and small females may also differ in their sensitivity to SP, for example through differences in SP receptor expression, or have different levels of insulin signalling, which can influence remating propensity (Wigby *et al*. 2011). Another non‐mutually exclusive explanation is that because large females produce eggs at a faster rate (Lefranc & Bundgaard 2000; Pitnick & García–González 2002; Byrne & Rice 2006; Lüpold *et al*. 2011) and females deplete sperm as they lay eggs (Manning 1962), large females might deplete their sperm and SP reserves more quickly than small females, thus decreasing the strength of the SP response more quickly. Females may have evolved mechanisms to match remating rates to fecundity in such a way that maintains fertility, via condition‐dependent modification of responses to Sfps. A further possibility – again not mutually exclusive to those above – is that females respond strategically to their developmental environment. Females that develop at low densities (i.e. large adult females) might mate more frequently to limit the risk of potentially not encountering a mate when they are sperm depleted. In contrast, females developing in high‐density environments might have mating patterns – marked by higher resistance to remating – consistent with being able to find a mate whenever sperm reserves are low.

We observed differences in mating duration between treatments that were influenced by both male and female sizes, with small males and large females generally mating for longer and large males showing a bigger difference between large and small females (see Data S1 and Fig. S1). The significance of mating duration in relation to Sfp transfer is hard to assess. This study and previous work (Sirot, Wolfner & Wigby 2011) indicate that there is not a consistent relationship between mating duration and Sfp quantity transferred. Moreover, recent evidence that males can tailor the composition of specific Sfps in their ejaculates in response to the mating environments means that there cannot possibly be consistent correlations between mating duration for every Sfp, because individual Sfp titres can vary independently (Sirot, Wolfner & Wigby 2011). We do not know whether males of different sizes transfer Sfps at different rates – for example, whether the generally shorter matings of large males in this study indicate more rapid Sfp transfer by large males – but this could be tested in future studies using a time series of interrupted matings (Gilchrist & Partridge 2000). Large variation in the rate and timing of Sfp transfer would have the potential to influence the detection of Sfps in the female reproductive tract measured at 25 min after the start of mating. However, given that the maximum differences in mating duration between treatments in this study are around 5 min (Fig. S1), there would likely be negligible effect on SP, because little SP dissipates from the reproductive tract within the first hour post‐mating (Sirot *et al*. 2009). Although OV dissipates more rapidly after mating (Sirot *et al*. 2009), several studies indicate that patterns of mating duration do not consistently correlate with OV levels in the female reproductive tract (Wigby *et al*. 2009; Sirot, Wolfner & Wigby 2011), suggesting that variation in mating duration is unlikely to explain the patterns of OV measured in females in general, despite the correlations found in this study. However, future research should investigate whether male or female developmental environment can influence the rate of Sfp transfer to, or movement from, the female reproductive tract.

## Conclusions

Environmental conditions during development have important consequences for many morphological, physiological, behavioural and life‐history traits, including those central to reproduction (Monaghan 2008). Previous studies have demonstrated that high densities or poor larval nutrition result in small body size in *D. melanogaster* (Miller & Thomas 1958), which, in turn, influences reproductive rates, mating patterns and female post‐mating responses (Partridge & Farquhar 1983; Pitnick 1991; Bangham, Partridge & Chapman 2002; Amitin & Pitnick 2007; Long *et al*. 2009; Zikovitz & Agrawal 2013). Our findings help to explain these previously described patterns by revealing how larval density mediates adult male Sfp production and allocation patterns, and female responses. Key challenges for the future include (i) determining to what extent sperm numbers and Sfp quantity are coupled or can be adjusted independently in males of varying condition, (ii) determining how common and predictable effects of condition and the perception of future competition levels are on Sfps are across taxa, (iii) exploring the physiological and neuronal mechanisms that underlie the ability of males and females to match their reproductive behaviours to their own body condition, that of their mates and the perceived competitive environment and (iv) revealing the consequences of developmental environment‐mediated reproductive patterns for the fitness of individuals and the strength and form of sexual selection in populations.

## Supporting information


**Lay Summary**

**Fig. S1.** First mating duration (mean ± SE) in response to male and female size (A, C) and the size of rival male (B).
**Fig. S2.** Courtship and rejection behavior.

**Data S1.** Statistics for experimental blocks, days, and ELISA plates.
