# Supplementary material for: Developmental environment mediates male seminal protein investment in Drosophila melanogaster
Source: Funct Ecol. 2015 Aug 20;30(3):410–9. doi: 10.1111/1365-2435.12515 (PMC4974917; doi:10.1111/1365-2435.12515)
Supplement: Supplementary file 1 — Lay Summary [file FEC-30-410-s001.pdf]

Functional Ecology
British Ecological Society

Developmental environment mediates male seminal protein investment in Drosophila melanogaster

Stuart Wigby, Jennifer C. Perry, Yon-Hee Kim, and Laura K. Sirot

Although genes influence how an animal looks and behaves, these characteristics are also shaped by environmental factors. For example, in some reptiles, genetically identical embryos can develop into either sex depending on the incubation temperature of the egg. Similarly striking is that whether a female bee develops into a worker or queen is determined by the food she is given as a larva, rather than by her genes. Scientists now understand that an animal's behavior and appearance are shaped by complex interactions between genes and environment.

In this study we used fruit flies to investigate whether intensity of competition for food early in life affects mating and reproduction in adulthood. In particular, we were interested in proteins that males transfer in their seminal fluid to females at mating, because we know from previous studies that these proteins – especially one named "sex peptide" – are important for male and female reproductive success. Flies that we reared at high densities developed into smaller adults – probably because they encountered more competition for food as juveniles – and, as expected, we discovered that small adult males had reduced stored reserves of seminal proteins and were slower to mate with females than large (low-density reared) males. However, small males transferred almost as much sex-peptide to females as did large males, and so invested a much greater relative proportion of their stored reserves. Small males might transfer so much of their sex-peptide reserves because they tend to get fewer opportunities to mate, so they hold less back, or because their highly competitive early life environment primes them for intense male-male competition as adults. We also found that large females re-mated more quickly than small females, which could be because they are fast reproducers and therefore need to replenish sperm reserves more frequently, or because their less competitive early life environment primes them to take mating opportunities more readily (e.g., in case there is a shortage of males). Overall, our results suggest that early life environment has important consequences for mating and reproduction in both male and female flies, and suggests that this is a topic ripe for further research.

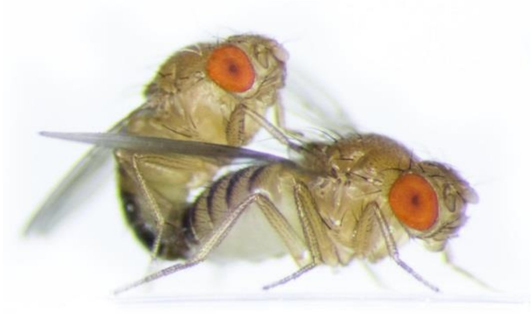

Image provided by authors.
