# Supplementary figures and images for: Developmental environment mediates male seminal protein investment in Drosophila melanogaster
Source: Funct Ecol. 2015 Aug 20;30(3):410–9. doi: 10.1111/1365-2435.12515 (PMC4974917; doi:10.1111/1365-2435.12515)

Supplemental Fig 1

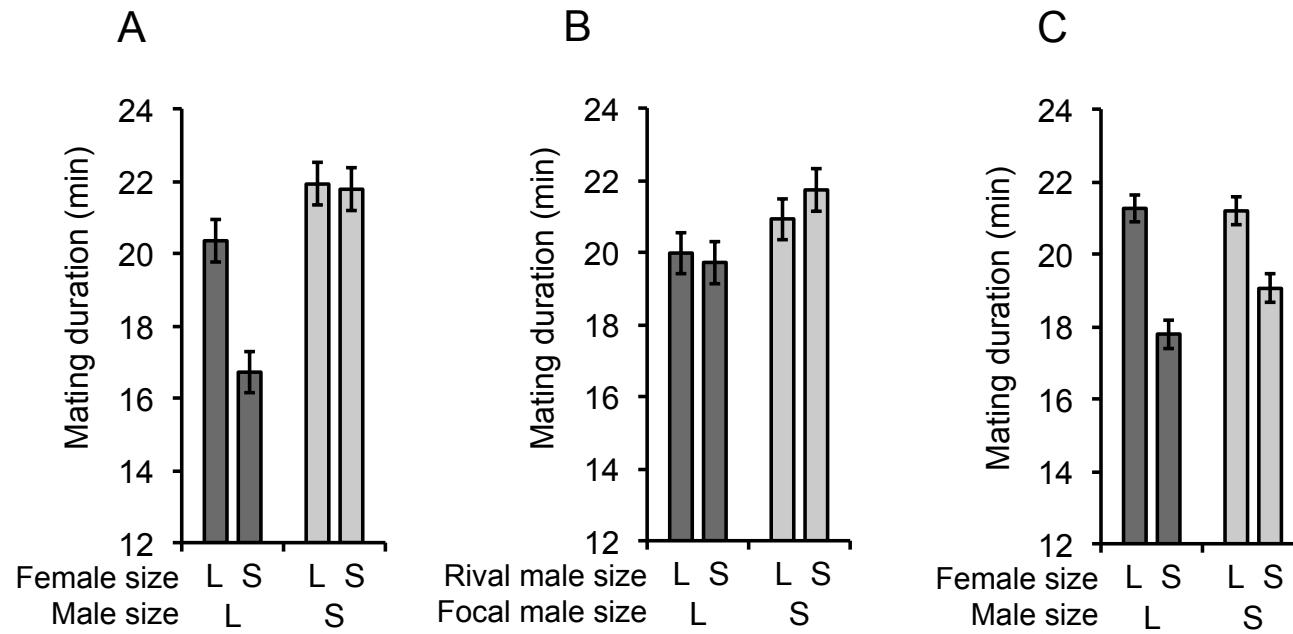

Supplemental Figure 2

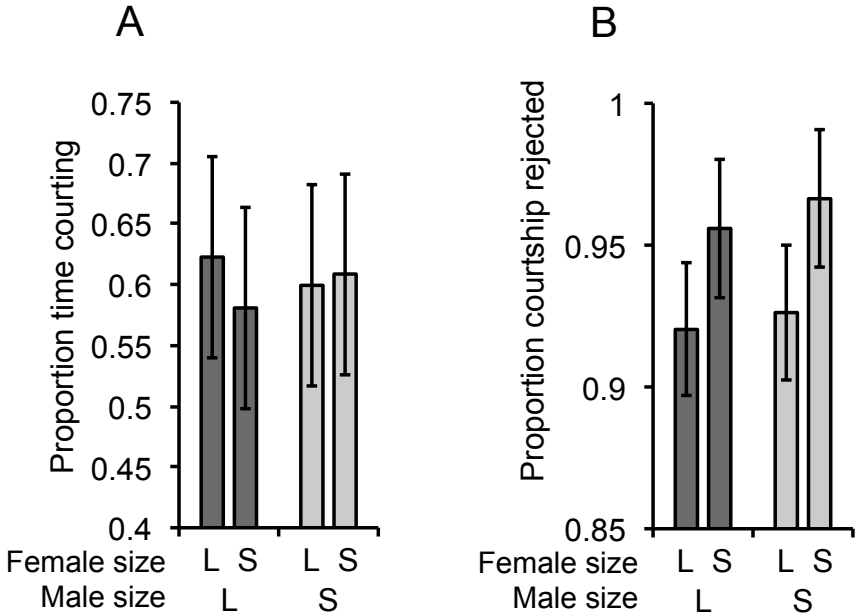

Supplement: Supplementary file 2 — Fig. S1. First mating duration (mean ± SE) in response to male and female size (A, C) and the size of rival male (B). Fig. S2. Courtship and rejection behavior. [file FEC-30-410-s002.pdf]
