# Supplementary material for: Developmental environment mediates male seminal protein investment in Drosophila melanogaster
Source: Funct Ecol. 2015 Aug 20;30(3):410–9. doi: 10.1111/1365-2435.12515 (PMC4974917; doi:10.1111/1365-2435.12515)
Supplement: Supplementary file 3 — Data S1. Statistics for experimental blocks, days, and ELISA plates. [file FEC-30-410-s003.docx]

**Supplemental material**

**Developmental environment mediates male seminal protein investment in *Drosophila melanogaster***

**Stuart Wigby^1^*, Jennifer C. Perry^1,2^, Yon-Hee Kim^1^, and Laura K. Sirot^3^***

* Authors contributed equally.

Addresses

1. Edward Grey Institute, Department of Zoology, University of Oxford, South Parks Road, OX1 3PS

2 Jesus College, University of Oxford, Turl Street, Oxford OX1 3DW United Kingdom

3. Department of Biology, College of Wooster, Wooster, OH, 44691, USA

**Correspondence:** SW, [stuart.wigby@zoo.ox.ac.uk](mailto:stuart.wigby@zoo.ox.ac.uk)

Tel: 01865 271 161 Fax: 01865 271168

**Emails:**

JCP: [jennifer.perry@zoo.ox.ac.uk](mailto:jennifer.perry@zoo.ox.ac.uk)

YHK: [yonheekim91@gmail.com](mailto:yonheekim91@gmail.com)

LKS: [lsirot@wooster.edu](mailto:lsirot@wooster.edu)

**Supplemental results**

**Mating duration.**

Combining p-values across experiments (Sokal & Rohlf 1995) revealed that mating duration is subject to a strong interaction between male and female sizes (p = 0.0001). The main effects of female (p <0.0001) and male size (p < 0.0001) were also significant. In Experiment 1, mating duration was strongly influenced by the interaction between male and female, as well as by block (male size, F_1,350_ = 63.95, p < 0.0001; female size, F_1,350_ = 21.05, p < 0.0001; male * female, F_1,350_ = 17.84, p < 0.0001; block, F_1,350_ = 5.05, p = 0.025; Supplemental Figure 1A). Mating duration was longer with large as compared to small females, but only with large males; there was no effect of female size on mating duration with small males. In Experiment 2 there was no significant interaction between focal male size and rival size (F_1,208_ = 1.24, p = 0.27) and no effect of rival size (F_1,209_ = 0.38, p = 0.54), but mating duration was significantly longer for small males (F_1,210_ = 9.89, p = 0.0019; Supplemental Figure 1B). In Experiment 3, matings with large females were of longer duration than with small females (F_1,356_ = 51.69, p < 0.0001), with no significant effect of male size (F_1,356_ = 2.43, p = 0.12; Supplemental Figure 1B). Although the interaction between male and female size was not significant (F_1,356_ = 2.80, p = 0.095), the trend was similar to that of Experiment 1, in that the difference in copulation duration between large and small females was less for small males compared to large males. Day number did not have a significant effect on mating duration (F_1,356_ = 0.22, p = 0.64), but there was significant variation between blocks (F_2,356_ *=* 6.46, p = 0.0018).

**REPEATABILITY OF SFP MEASURES**

Repeatability was estimated using the intraclass correlation coefficient and 95% confidence interval for duplicate measures of Sfps in the same biological sample. The repeatabilities of the SP and OV measures are as follows:

Experiment 1: OV in female reproductive tracts after mating: ICC: 0.95 95% CI: 0.94-0.96

Experiment 1: SP in female reproductive tracts: ICC: 0.90 95% CI: 0.86-0.91

Experiment 2: SP in female reproductive tracts after mating: ICC: 0.91 95% CI: 0.88-0.93

Experiment 2: SP in male reproductive tracts after mating: ICC: 0.80 95% CI: 0.72-0.86

**STATISTICS FOR EXPERIMENTAL BLOCKS, DAYS, AND ELISA PLATES**

***Seminal protein production***

In Experiment 1, there was significant variation between ELISA plates in the quantity of SP detected, but no block effect (ELISA plate, F_1,35_ = 23.87, p< 0.0001; block F_1,34_ = 2.38, p = 0.13).

In Experiment 2 there was significant variation among ELISA plates in amount of SP detected (F_4,93_ = 7.40, p <0.0001). For OV, there were no significant block or ELISA plate effects (Experiment 1; block F_1,37_ = 0.35, p = 0.56; ELISA plate, F_1,36_ = 0.025, p = 0.87).

***Seminal Fluid Protein Allocation to Females***

*Absolute amount of Sfps transferred during mating*

For SP there was significant variation among ELISA plates (F_4,278_ = 26.50, p < 0.0001) in Experiment 1. In Experiment 2, ELISA plate was treated as a random factor (because there were >7 levels (Bolker *et al.* 2009; Zikovitz & Agrawal 2013)), hence p-values are not reported. For OV, there were significant ELISA plate effects (large females, F_1,131­_ = 19.21, p < 0.0001; small females, F_1,140_ = 9.16, p < 0.0001).

*Proportion of sex peptide transferred during mating*

Neither male nor female ELISA plates were significant factors in the proportion of SP transferred to females (male ELISA plate, F_4,91_ = 1.49, p = 0.21; female ELISA plate, F_4,95_ = 1.89, p = 0.12).

***Male and Female Mating Rates***

*Virgin females*

There was significant variation between blocks in those experiments conducted in blocks (Experiment 1: F_1,359_ = 5.02, p = 0.026; Experiment 3; F_2,359_ = 10.06, p < 0.0001). Latency to mating decreased with day in Experiment 3 (day F_1,359_ = 33.99, p < 0.0001) which is likely a result increased mating receptivity with female age.

*Experiment 3: Remating in previously mated females*

There were no significant day effects (χ_1_ = 1.51, p = 0.22) or block effects (χ_2_ = 1.63, p > 0.1).

*Experiment 4: Courtship and rejection*

There were no day effects for courtship or rejection rates by previously mated females (Courtship, Day, F_1,75_ = 0.036, p = 0.85; Rejection: Day, F_1,69_ = 0.48, p = 0.49).

**Supplemental Figure Legends**

Supplemental Figure 1. First mating duration (mean ± S.E.) in response to male and female size (A, C) and the size of rival male (B). A) Experiment 1, B) Experiment 2, C) Experiment 3.

Supplemental Figure 2. Courtship and rejection behavior. A) male courtship rate and B) female rejection of male courtship in response to male and female size (mean ± S.E.).

References:

Bolker, B.M., Brooks, M.E., Clark, C.J., Geange, S.W., Poulsen, J.R., Stevens, M.H.H. & White, J.-S.S. (2009) Generalized linear mixed models: a practical guide for ecology and evolution. *Trends in Ecology & Evolution,* **24,** 127-135.

Sokal, R.R. & Rohlf, F.J. (1995) Combining probabilities from tests of significance. *Biometry: the principles and practice of statistics in biological research***,** 794-797.

Zikovitz, A.E. & Agrawal, A.F. (2013) The condition dependency of fitness in males and females: the fitness consequences of juvenile diet assessed in environments differing in key adult resources. *Evolution,* **67,** 2849-2860.
